# Fat Embolism and Nonconvulsive Status Epilepticus

**DOI:** 10.1155/2018/5057624

**Published:** 2018-12-20

**Authors:** Yanetsy Olivera Arencibia, Mai Vo, Jennifer Kinaga, Jorge Uribe, Gloria Velasquez, Mario Madruga, S. J. Carlan

**Affiliations:** ^1^Department of Medicine, Orlando Regional Healthcare, Orlando, Florida, USA; ^2^Division of Academic Affairs and Research, Orlando Regional Healthcare, Orlando, Florida, USA

## Abstract

Fat embolism syndrome (FES) typically occurs following orthopedic trauma and may present with altered mental status and even coma. Nonconvulsive status epilepticus is an electroclinical state associated with an altered level of consciousness but lacking convulsive motor activity and has been reported in fat embolism. The diagnosis is clinical and is treated with supportive care, antiepileptic therapy, and sedation. A 56-year-old male presented with altered mental status following internal fixation for an acute right femur fracture due to a motor vehicle accident 24 hours earlier. Continued neuromonitoring revealed nonconvulsive status epilepticus. Magnetic resonance imaging of the brain showed multiple bilateral acute cerebral infarcts with a specific pattern favoring the diagnosis of fat embolism syndrome. He was found to have a significant right to left intracardiac shunt on a transesophageal echocardiogram. He improved substantially over time with supportive therapy, was successfully extubated on day 6, and discharged to inpatient rehabilitation on postoperative day 15. Fat embolisms can result in a wide range of neurologic manifestations. Nonrefractory nonconvulsive status epilepticus that responds to antiepileptic drugs, sedation, and supportive therapy can have a favorable outcome. A high index of suspicion and early recognition reduces the chances of unnecessary interventions and may improve survival.

## 1. Introduction

Fat embolism syndrome (FES) consists of respiratory manifestations, cerebral symptoms, and cutaneous petechiae as a result of fat emboli causing tissue damage through vascular occlusion, ischemia, and activation of coagulation and systemic inflammatory response [1, 2]. It can occur following pelvic or long bone fractures as well as after orthopedic operations or any clinical condition where fat is manipulated. Rare cases are nontrauma-related. The incidence of FES among patients with long-bone fractures is low, ranging from 2 to 5% [1] and the mortality rates are as high as 10% [3]. The diagnosis is mainly clinical and there is no definitive treatment. Respiratory involvement occurs early. Up to 75% of the patients present with sudden dyspnea, tachypnea, and hypoxemia of varying degrees of severity [3, 4]. Cutaneous manifestations are present on approximately 50% of cases. Neurological manifestations are present in 85% of patients with FES and can include confusion, clouding of consciousness, seizures, focal deficits, and even coma [5]. Nonconvulsive status epilepticus (NCSE) is an electroclinical state associated with an altered level of consciousness but lacking convulsive motor activity lasting at least 30 minutes [6]. Electroencephalographic confirmation of the seizure is necessary for the diagnosis of NCSE. Treatment is not straightforward and depends on many elements, including the etiology, electroencephalogram (EEG) findings, and the clinical status of the patient [7].

We report a case of a male with nonconvulsive status epilepticus as first manifestation of fat embolism syndrome in the setting of orthopedic fixation of an acute right femur fracture after a motor vehicle accident.

## 2. Case Presentation

**Figure 1:**
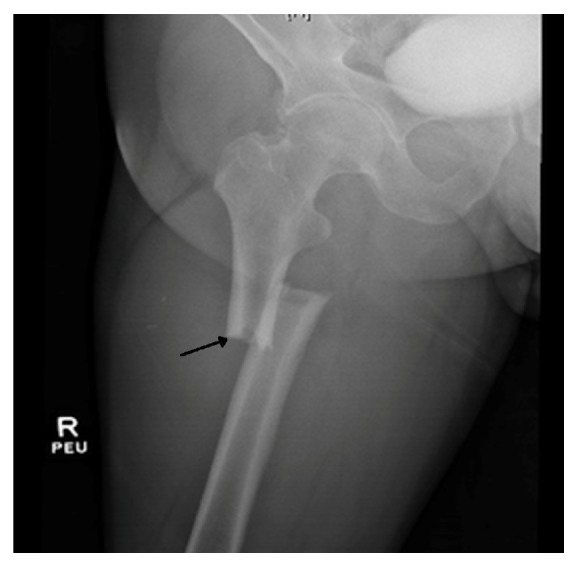
Right Femur X-Ray showing acute femur fracture (black arrow).

A 56-year-old male with a history of hypothyroidism and hyperlipidemia was admitted to the hospital after a motor vehicle accident where he sustained a closed right subtrochanteric femur fracture with 4 cm of foreshortening [Figure 1]. On admission his hemoglobin was 14 g/dL, white blood cell count 7.7 x 10^3^/*μ*L, platelet count 220 x 10^3^/*μ*L, and metabolic panel normal with the exception of plasma venous glucose of 120 mg/dL. His urine drug screen was negative and he was alert with no mental status disturbance. He was taken to the operative room within the first few hours of admission and underwent central medullary nailing without any intraoperative complications. Immediately post-op he was drowsy from the anesthesia but following commands properly. On postoperative day 1, he was found to be very lethargic and confused with altered mental status. He was normotensive, with temperature of 37.7 C, heart rate of 109 beats per minute, respiratory rate of 24 breaths per minute, and pulse oximetry showing 92% oxygen saturation on room air. On examination he was very drowsy but following basic commands. His right leg was immobilized with ecchymosis of the same leg noticed and distal extremity pulses were normal bilaterally. On neurologic examination, a right sided weakness of the upper extremity was appreciated, but muscle tone was normal, and reflexes were preserved. The Glasgow score was 10/15. His skin was warm and dry with no evidence of petechial rash at that time. His pupils were reacting to light, and his fundoscopy was within normal limits. His routine investigations revealed no significant derangements in the complete blood count and renal or liver functions. There was no evidence of thrombocytopenia. The differential diagnosis included narcotic overdose since he was getting pain medications, fat embolism, postoperative delirium, acute cerebrovascular accident (CVA), and sepsis.

**Figure 2:**
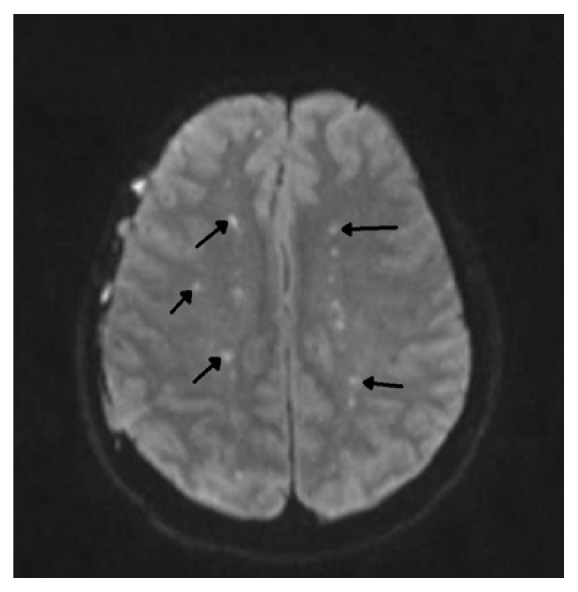
Brain MRI revealed multiple bilateral acute cerebral infarcts (black arrows) with restricted diffusion areas in a “starfield” pattern favoring fat emboli.

**Figure 3:**
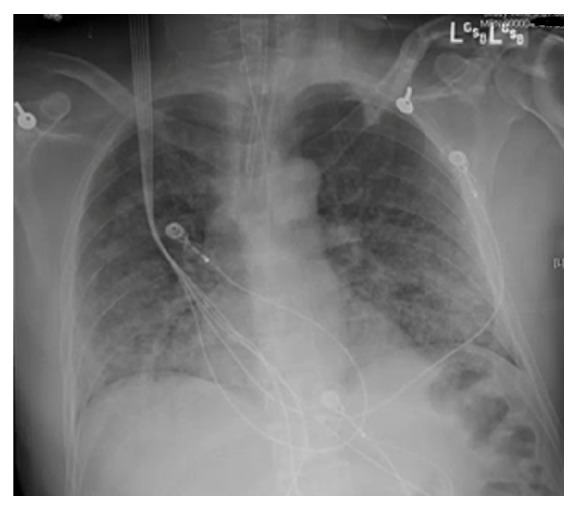
CXR showed diffuse bilateral infiltrates in a “snowstorm” pattern.

**Figure 4:**
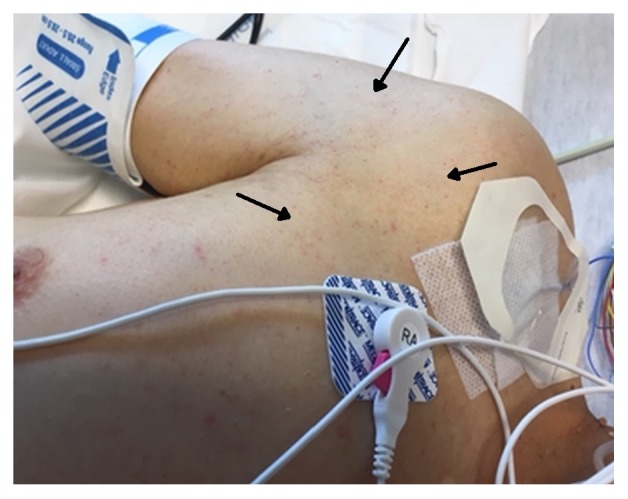
Anterior chest wall petechial rash (black arrows).

**Figure 5:**
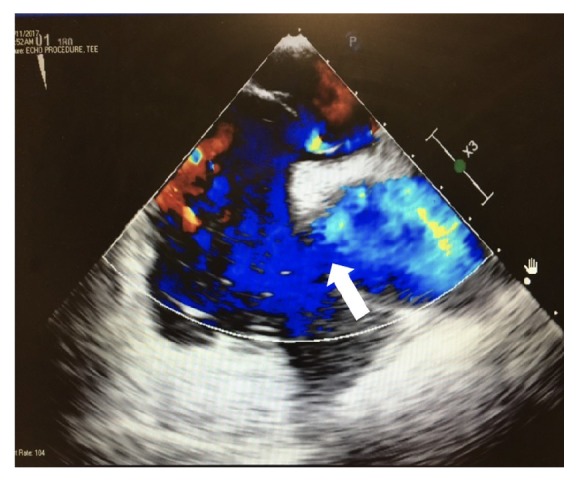
Transesophageal echocardiogram showing significant R to L shunt (white arrow).

**Figure 6:**
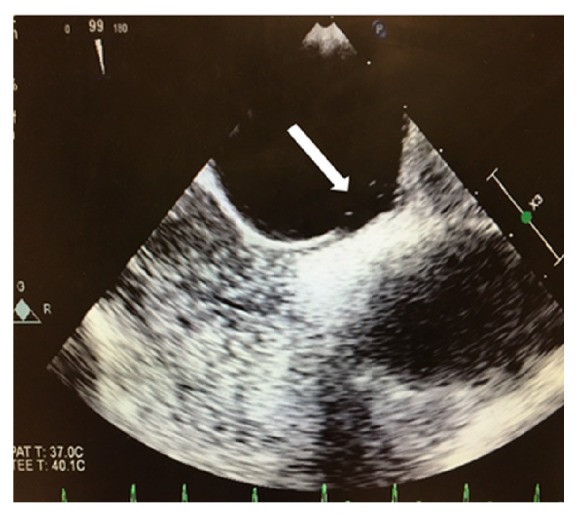
White arrow showing 3 bubbles crossing the atrial septum on transesophageal echocardiogram.

One dose of naloxone, 0.4 mg intravenously, was given without improvement. He was placed under stroke alert. No acute intracranial hemorrhage was seen on noncontrast computerized tomographic (CT) scan of the head and his electrocardiogram (EKG) was normal. A right lower extremity artery duplex Doppler examination was normal and chest CT angiogram was negative for pulmonary embolism. As the day progressed he became more obtunded requiring an intensive care unit (ICU) admission and endotracheal intubation for airway protection. Magnetic resonance imaging (MRI) of the brain revealed multiple bilateral acute cerebral infarcts with restricted diffusion areas in a “starfield” pattern favoring the diagnosis of cerebral fat embolism [Figure 2]. He remained unresponsive and continued neuromonitoring revealed multifocal epileptiform discharges and continual ictal discharges from the right anterior quadrant with contralateral spread consistent with nonconvulsive status epilepticus. He was immediately started on midazolam drip at 5 mg/hour, loaded with 20mg/kg of fosphenytoin maintenance dose of 100 mg every 8 hrs. Due to failure on ictal discharge suppression after initial intervention, midazolam drip was titrated up and a second antiepileptic drug was started, levetiracetam 1000 mg every 8 hrs. Nonconvulsive status epilepticus resolved after 72 hours of the diagnosis. Lumbar puncture was not considered necessary for his management. Within the first 48 hours of ICU admission he developed a bilateral patchy opacity in a “snowstorm” pattern on chest X-Ray [Figure 3] and his BNP (brain natriuretic peptide) was 24 (normal 0-100 pg/mL). There was a petechial rash noted on his anterior chest [Figure 4]. Further workup demonstrated a patent foramen ovale (PFO) with a significant right to left shunt [Figure 5] by transesophageal echocardiogram (TEE). There was also a positive bubble study [Figure 6]. A diagnosis of fat embolism syndrome was made. He improved with antiepileptics and supportive care. He was extubated on postoperative day 6 with significant mental status improvements; however, he had dense right-sided weakness, which improved significantly after 3 more days. He was discharged to inpatient rehabilitation on postoperative day 15.

## 3. Discussion

There are only two previous reports of patients with EEG confirmed NCSE complicating fat embolism [8, 9]. This means it is either underreported, underdetected, or extremely unusual. Since continuous EEG monitoring is a necessary component of the diagnosis, it is likely that NCSE is underdetected and therefore underreported. The presence of NCSE may be important, however, since the patients' response to antiepileptic therapy and central sedation may be a variable in the prognosis. Nonetheless, the two previous cases of NCSE and ours share similarities. All three cases occurred proximal to an orthopedic incident and were characterized by cranial MRI showing small scattered punctate areas of restricted diffusion consistent with acute intracranial ischemic infarcts. The EEG in all three cases displayed epileptiform discharges consistent with NCSE and the treatment included intubation, sedation, and multiple antiepileptic medications. One case, however, is notable for its response to treatment. An 82-year-old woman could not be weaned from her heavy sedation and died [9]. An autopsy confirmed her cause of death was FES suggesting that super-refractory nonconvulsive status epilepticus secondary to fat embolism may be a category with a much worse prognosis. Of note was her FES followed an elective knee prosthesis surgery while the other 22-year-old and our 56-year-old involved the male gender with traumatic fractured femurs. In addition, our patient had already undergone an internal fixation procedure while the 22-year-old's FES occurred before orthopedic surgery. One other notable difference between the two patients that survived the FES was the etiology of the ischemic brain lesions. Our patient had a demonstrable intracardiac shunt creating a likely route for fat emboli to the brain. The 22-year-old, however, had no apparent intracardiac or intrapulmonary shunt on imaging and examination. This suggests that an alternative pathophysiological mechanism can result in FES. In fact, similar brain lesions can occur with hypoxic encephalopathy regardless of the etiology, but the 22-year-old was intubated prophylactically. The biochemical theory [9] states that severe hypoxia associated with FES-induced respiratory failure can create ischemic brain infarcts, but this patient was never hypoxic according to the report. It is possible that plasma mediators released during the acute phase of trauma resulted in lipolysis and a subsequent inflammatory response in various tissues including the brain.

Whatever the pathophysiology the commonalities of both cases that survived include the male gender, femur fracture, and, most importantly, the absence of a super-refractory variety of NCSE. If, in fact, the response to treatment of NCSE is a reproducible variable for predicting survival of patients with encephalopathy-associated FES then continuous EEG becomes an essential and critical element of management. NCSE refractory to antiepileptic therapy and sedation may change the prognosis and expectations during counseling to the victim's family. Finally, this case is unique in the presence of FSE likely achieved through a verified PFO.

In summary, the neurologic manifestations of FES may include NCSE, which suggests continuous neuromonitoring through the initial insult is indicated. If the NCSE is nonrefractory there may be a more favorable prognosis than refractory NCSE and counseling can be adjusted accordingly. Early diagnosis of FES in a patient without head trauma may be difficult even with suspicious neurologic decline. Without continuous EEG monitoring NCSE would be easily overlooked.
